# Catalytic properties of Co_3_O_4 _nanoparticles for rechargeable Li/air batteries

**DOI:** 10.1186/1556-276X-7-47

**Published:** 2012-01-05

**Authors:** Kwan Su Kim, Yong Joon Park

**Affiliations:** 1 Department of Advanced Materials Engineering, Kyonggi University, San 94-6, Yiui-dong, Yeongtong-gu, Suwon, Gyeonggi-do, 443-760, Republic of Korea

**Keywords:** composites, nanostructures, chemical synthesis, electrochemical properties.

## Abstract

Three types of Co_3_O_4 _nanoparticles are synthesized and characterized as a catalyst for the air electrode of a Li/air battery. The shape and size of the nanoparticles are observed using scanning electron microscopy and transmission electron microscopy analyses. The formation of the Co_3_O_4 _phase is confirmed by X-ray diffraction. The electrochemical property of the air electrodes containing Co_3_O_4 _nanoparticles is significantly associated with the shape and size of the nanoparticles. It appears that the capacity of electrodes containing villiform-type Co_3_O_4 _nanoparticles is superior to that of electrodes containing cube- and flower-type Co_3_O_4 _nanoparticles. This is probably due to the sufficient pore spaces of the villiform-type Co_3_O_4 _nanoparticles.

## Introduction

A significant increase in the energy density of rechargeable batteries is required to satisfy the demands of vehicular applications and energy storage systems. One approach to solving this problem is the introduction of a new battery system having a higher energy density. Li/air batteries are potential candidates for advanced energy storage systems because of their high storage capability [1-3]. They do not store a 'cathode' in the system, which allows for a higher energy density than any other commercial rechargeable batteries. Instead, oxygen from the environment is reduced by a catalytic surface inside the air electrode. Thus, catalysts are key materials that affect the capacity, cycle life, and rate capability of such batteries.

In this study, the Co_3_O_4 _nanoparticles of various shapes and structures were tested as catalysts of air electrodes for rechargeable Li/air batteries. Co_3_O_4 _with a spinel structure has attracted a considerable interest as a potential catalyst in various application fields [4-7]. In particular, this study was motivated by the notion that the catalytic efficiency of oxides is highly dependent on their morphology, size, and crystal structure [8,9]. Herein, three types of Co_3_O_4 _of various shapes and morphologies were synthesized, and the electrochemical properties of the air electrodes containing Co_3_O_4 _nanoparticles were characterized.

## Experimental details

Three types of Co_3_O_4 _nanoparticles were prepared by a hydrothermal reaction using cobalt nitrate (cube type, flower type) and cobalt chloride (villiform type), considering previous reports [10,11]. Surfactants such as urea were also added to obtain nanosized particles. X-ray diffraction [XRD] patterns of powders were measured using a Rigaku X-ray diffractometer (Rigaku Corporation, Tokyo, Japan). The microstructure of the powder was observed by field-emission scanning electron microscopy [FE-SEM] (JEOL-JSM 6500F, JEOL Ltd., Akishima, Tokyo, Japan) and field-emission transmission electron microscopy [FE-TEM] (JEOL-JEM 2100F JEOL Ltd., Akishima, Tokyo, Japan). The electrochemical performance of the air electrode containing Co_3_O_4 _nanoparticles was examined using a modified Swagelok cell, consisting of a cathode, a metallic lithium anode, a glass fiber separator, and an electrolyte of 1 M LiTFSI in EC/PC (1:1 vol.%). The cathode contained carbon (Ketjen black EC600JD, Akzo Nobel, Amsterdam, The Netherlands; approximately 1420 m^2^·g^-1^), catalysts (Co_3_O_4 _nanoparticles), and a binder (PVDF; Sigma-Aldrich, St. Louis, MO, USA). The molar ratio of carbon to catalysts was adjusted to 95:5. The binder accounted for 20 wt.% of the total electrode. The cells were assembled in an Ar-filled glove box and subjected to galvanostatic cycling using a WonATech (WBCS 3000, Seocho-gu, Seoul, Korea) charge-discharge system. Experiments were carried out in 1 atm of O_2 _using an air chamber.

## Results and discussion

**Figure 1:**
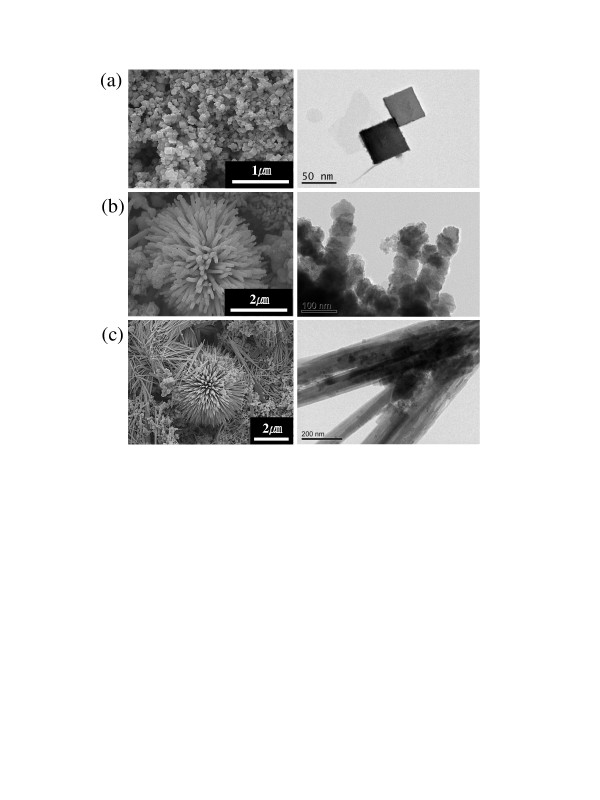
**SEM (left side) and TEM (right side) images of the Co_3_O_4 _nanoparticles**. (**a**) Cube type, (**b**) villiform type, and (**c**) flower type.

**Figure 2:**
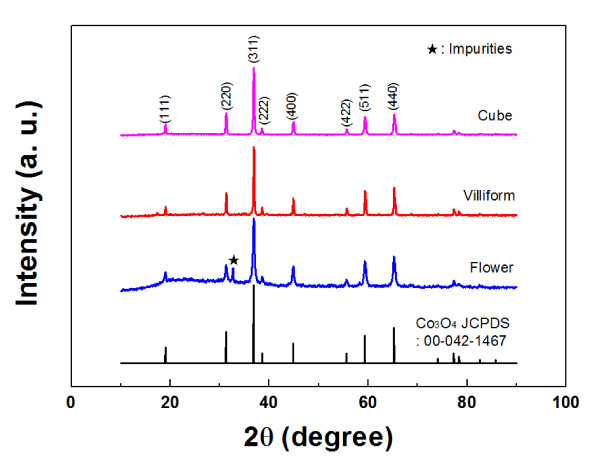
**XRD patterns of the Co_3_O_4 _nanoparticles and reference Co_3_O_4_**.

Scanning electron microscopy [SEM] and transmission electron microscopy [TEM] were employed to investigate the shapes of the samples (Figure 1). Cube-type Co_3_O_4 _nanoparticles have a homogeneous cubic morphology (Figure 1a). The length of the nanocube was around 200 nm, and the dominant exposed plane of the cube-type Co_3_O_4 _seemed to be {001}. The villiform-type Co_3_O_4 _particles were formed by a nucleus covered with numerous micrometer-sized nanorods. In comparison with the length, the diameter of the nanorod was very small (less than 100 nm). It is interesting that the villiform-type Co_3_O_4 _has a rough surface. As shown in the TEM image (Figure 1b), the nanorods seemed to be stacked with smaller nanoparticles with a diameter of approximately 80 nm. The flower-type Co_3_O_4 _seemed to have a similar shape and size to those of the villiform-type Co_3_O_4_. However, the nanorods of the flower-type Co_3_O_4 _had a sharper end, smoother surface, and smaller diameter than those of the villiform-type Co_3_O_4_. Moreover, in contrast with the villiform-type Co_3_O_4_, the nanorods of the flower-type Co_3_O_4 _particles were almost separated during the preparation process for the TEM experiments (Figure 1c). This implies that the flower-type Co_3_O_4 _particles may turn to the nanorod type during the electrode fabrication process because of vigorous mixing in making a slurry. The crystallinity of the three types of Co_3_O_4 _nanoparticles was investigated by XRD. As shown in Figure 2, all XRD peaks of the cube-type Co_3_O_4 _nanoparticles can be indexed to the Co_3_O_4 _spinel phase, indicating a single-phase sample. Most diffraction peaks for villiform- and flower-type Co_3_O_4 _particles were also identical to those of the typical Co_3_O_4 _phase; however, small impurities could be detected in the diffraction patterns.

**Figure 3:**
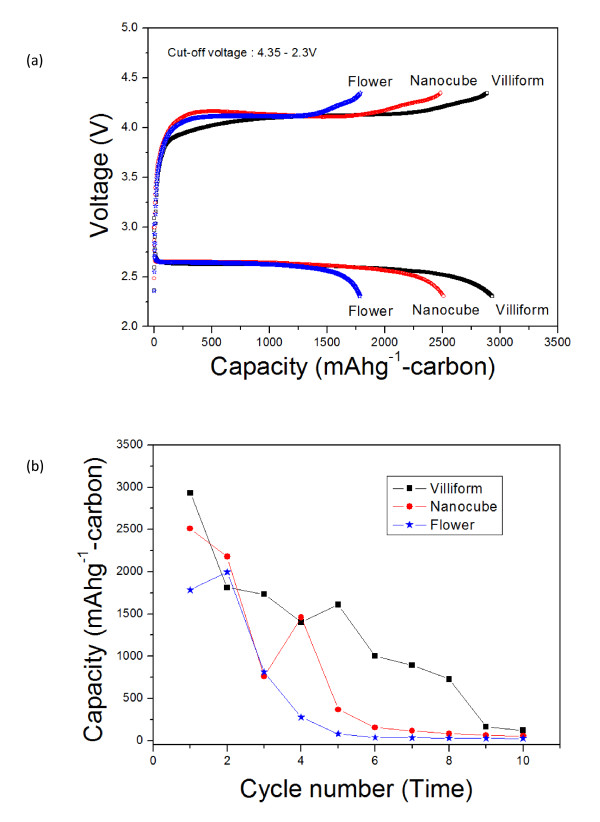
**Electrochemical properties of the air electrode containing Co_3_O_4 _nanoparticles**. Air electrode containing Co_3_O_4 _nanoparticles at a constant current density of 0.4 mA·cm^-2 ^(voltage range of 4.35 to 2.3 V). (**a**) Initial voltage profile and (**b**) cyclic performance.

The electrochemical properties of the air electrodes containing Co_3_O_4 _nanoparticles were characterized at a constant current density of 0.4 mA·cm^-2 ^at 30°C. Figure 3a shows the initial voltage profile of the electrodes containing the Co_3_O_4 _nanoparticles in the voltage range of 4.35 to 2.3 V. The discharge capacity shown in Figure 3 is based on the weight of carbon (Ketjen black) in the air electrode, which has generally been used for expressing the capacity of an air electrode [1,8,9,12]. The average charge and discharge voltages of the air electrode containing the Co_3_O_4 _nanoparticles were approximately 4.2 and 2.6 V, respectively. The initial discharge capacity of the electrode was highly dependent upon the type of Co_3_O_4 _nanoparticles. The electrode containing villiform-type Co_3_O_4 _nanoparticles showed a relatively higher initial discharge capacity (approximately 2, 900 mA h·g^-1^) than with the other electrodes. In contrast, the initial discharge capacities of the electrodes containing flower-type Co_3_O_4 _nanoparticles were just about 1, 800 mA h·g^-1 ^although they have a shape very similar to the villiform-type Co_3_O_4 _nanoparticles. As shown in Figure 3b, the cyclic performance of the air electrodes was not satisfactory. Actually, capacity fading has been a typical feature of all previous results about air electrodes [8,12,13]. It has been known that cycle degradation is associated with irreversible reaction products, which accumulate in the pores of the electrode at a discharged state [13,14]. It seems that the practical rechargeability of air electrodes has yet to be achieved before these can be put to practical use.

**Figure 4:**
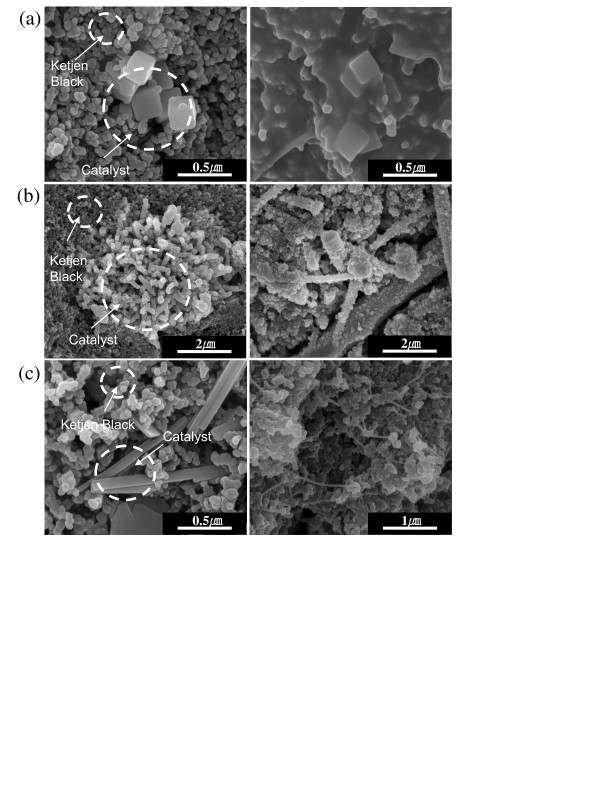
**SEM images of the air electrodes**. Air electrodes composed of Co_3_O_4 _nanoparticles, carbon (Ketjen black), and binder before the test and after discharge at 2.3 V. (**a**) Cube type, (**b**) villiform type, and (**c**) flower type.

After 10 cycles, the electrode was discharged to 2.3 V, and the surface was observed by SEM to investigate the morphology change during cycling. In the SEM images of the air electrodes before testing, the Co_3_O_4 _nanoparticles and carbon (Ketjen black) could be clearly identified (Figure 4). It was noticeable that the villiform-type Co_3_O_4 _nanoparticles maintained their shape during the electrode-fabrication process. However, the flower-type Co_3_O_4 _nanoparticles were almost separated to become the nanorod type. When they discharged to 2.3 V, it was observed that the surface of the electrode was homogenously covered with precipitates, which appeared to be reaction products such as lithium oxides, and lithium carbonates formed due to electrolyte decomposition [15,16]. These reaction precipitates could block the catalyst/carbon contact area, thereby preventing O_2 _intake and Li^+ ^delivery to the active reaction site and terminating the discharge process. According to previous reports [13,14], there was a strong correlation between average pore diameter and discharge capacity. Reaction precipitates are likely to be formed near active sites so that the micropore of a porous electrode would be easily sealed with precipitates of lithium oxides during discharge. Thus, securing enough space between catalytic active sites might increase the discharge capacity of the air electrode. The cube- and flower- (nanorod- in the electrode) type Co_3_O_4 _nanoparticles may be well covered with small carbon particles (Ketjen black) in the air electrode so that a sufficiently small pore space could be obtained. On the other hand, the villiform-type Co_3_O_4 _nanoparticles were composed of a nucleus covered with many nanorods of approximately 100 nm in size, which could offer enough space between active catalytic sites. Thus, a greater amount of lithium oxide precipitation may be needed to block the pore orifices and terminate the discharge process; this could be an explanation for the higher discharge capacity of the air electrode containing villiform-type Co_3_O_4 _nanoparticles in comparison with the air electrode containing other types Co_3_O_4 _nanoparticles.

## Conclusions

Cube-, flower-, and villiform-type Co_3_O_4 _nanoparticles were synthesized and introduced as catalysts for Li/air batteries. The electrochemical properties of the air electrodes containing Co_3_O_4 _nanoparticles were found to be highly dependent on the type of Co_3_O_4 _nanoparticles. The electrode containing villiform-type Co_3_O_4 _nanoparticles showed a higher discharge capacity than the electrodes containing other types of Co_3_O_4 _nanoparticles. This is likely due to the relatively sufficient pore space between active catalytic sites, which stores a large amount of reaction products.

## Abbreviations

EC: ethylene carbonate; FE-SEM: field-emission scanning electron microscopy; FE-TEM: field-emission transmission electron microscopy; LiTFSI: lithium bis(trifluoromethanesulfonyl)imide; PC: propylene carbonate; PVDF: polyvinylidene fluoride; XRD: X-ray diffraction.

## Competing interests

The authors declare that they have no competing interests.

## Authors' contributions

KS did the synthetic and characteristic works in this journal. YJ gave the advice and guided the experiment. All authors read and approved the final manuscript.
